# Coordination Polymers Containing 1,3-Phenylenebis-((1*H*-1,2,4-triazol-1-yl)methanone) Ligand: Synthesis and ε-Caprolactone Polymerization Behavior

**DOI:** 10.3390/molecules22111860

**Published:** 2017-10-29

**Authors:** Nestor J. Bello-Vieda, Ricardo A. Murcia, Alvaro Muñoz-Castro, Mario A. Macías, John J. Hurtado

**Affiliations:** 1 Department of Chemistry, Universidad de los Andes, Carrera 1 N° 18A-12, Bogotá 111711, Colombia; nj.bello1211@uniandes.edu.co (N.J.B.-V.); ra.murcia@uniandes.edu.co (R.A.M.); ma.maciasl@uniandes.edu.co (M.A.M.); 2 Grupo de Química Inorgánica y Materiales Moleculares, Universidad Autonoma de Chile, El Llano Subercaseaux 2801, Santiago 8910060, Chile; aemunozc@gmail.com; 3 Relativistic Molecular Physics (ReMoPh) Group, Universidad Andres Bello, Republica 275, Santiago, Chile

**Keywords:** triazole ligand, coordination polymers, initiators, ε-caprolactone polymerization

## Abstract

The reaction of isophthaloyl dichloride with 1*H*-1,2,4-triazole afforded the new ligand 1,3-phenylenebis(1,2,4-triazole-1-yl)methanone (**1**). A series of Co(II), Cu(II), Zn(II) and Ni(II) complexes were synthesized using **1** and then characterized by melting point analysis, elemental analysis, theoretical calculations, thermogravimetric analysis, X-ray powder diffraction, nuclear magnetic resonance, infrared and Raman spectroscopy. Experimental and computational studies predict the formation of coordination polymers (CPs). The cobalt and copper CPs and zinc(II) complex were found to be good initiators for the ring-opening polymerization of ε-caprolactone (CL) under solvent-free conditions. ^1^H-NMR analysis showed that the obtained polymers of CL were mainly linear and had terminal hydroxymethylene groups. Differential scanning calorimetry showed that the obtained polycaprolactones had high crystallinity, and TGA showed that they had decomposition temperatures above 400 °C. These results provide insight and guidance for the design of metal complexes with potential applications in the polymerization of CL.

## 1. Introduction

Currently, many researchers have focused on the synthesis of biodegradable polymers as there is great interest in designing environmentally friendly materials [1]. Polycaprolactone (PCL) is an important polymer used in agricultural, biomedical and environmental fields due to its tunable properties and biodegradability [2,3]. PCL is typically produced by the ring-opening polymerization of ε-caprolactone (CL) catalyzed by metal complexes [4,5,6,7]. One of the first catalysts used in this type of polymerization was titanium isopropoxide. This catalyst has a number of drawbacks, including high production costs and sensitivity to normal environmental conditions such as moisture and oxygen [3].

Other metal catalysts used for the ring-opening polymerization (ROP) of CL require initiators, which makes polymerization reactions more complex and expensive [8,9]. Some catalysts are toxic and can leave trace metals in the polymers. Additionally, special handling techniques, such as high-vacuum conditions and all-glass apparatus, are required for these catalysts, and these added difficulties make them impractical [6,10,11]. In view of this, it is very important to develop catalysts that are easy to prepare, are non-toxic and can catalyze the desired reaction under normal atmospheric conditions with adequate catalyst loading and at moderate temperatures. In this context, we have reported the synthesis, characterization and application of PCL catalysts using zinc and chromium cations. The ROP was carried out under solvent-free conditions with a monomer-complex ratio of 490:1. The synthesized PCL was highly crystalline (61%) and had a decomposition temperature above 300 °C [12,13].

Recently, the use of ligands with nitrogen and oxygen with different types of metals has been explored to drive the polymerization of caprolactone [14]. Magnesium and calcium complexes with mixed ligands showed low activity, with conversions between 5% and 40% after 12 h at 60 °C with a monomer-complex ratio of 200:1 [15]. Scorpionate ligands with azolic rings have been studied with very promising results. Complexes using these types of ligands with magnesium produce polymers with conversions of 98% at room temperature at a monomer-complex ratio of 500:1 [16]. As far as we know, there are very few reports of transition metal complexes with triazole ligands being used for CL polymerization [7,17]. In our search for related polymerization catalysts, we synthesized and characterized a series of new neutral Co(II), Cu(II), Zn(II) and Ni(II) coordination polymers containing 1,3-phenylenebis(1,2,4-triazol-1-yl)methanone ligands and their behavior as catalysts to polymerize CL. The polymers of PLC obtained were highly crystalline and had decomposition temperatures above 400 °C.

## 2. Discussion

### 2.1. Synthesis of 1,3-Phenylenebis(1,2,4-triazol-1-yl)methanone *(**1**)*

**Scheme 1:**
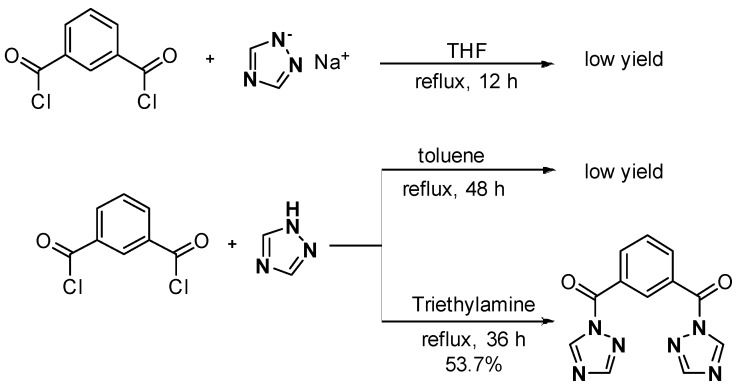
Synthesis of ligand **1**.

Initially, we attempted to synthesize the compound through the addition of isophthaloyl dichloride and 1*H*-1,2,4-triazole with anhydrous tetrahydrofuran and toluene as co-solvents. This procedure did afford some ligand, but the yield was very low. It was then decided to first synthesize the sodium salt of the triazole using a reported method [18]. This method consisted of reacting the triazole and a stoichiometric amount of NaOH in methanol for 4 h at room temperature. Then, the reaction mixture was cooled to −15 °C, and the salt was obtained as a hygroscopic white solid. The solid was isolated by filtration and washed with cold methanol and ethyl ether. This salt was later used as precursor for the reaction and did not require an exogenous base. However, the yield remained low. Finally, the ligand was obtained by the reaction of 1*H*-1,2,4-triazole and isophthaloyl chloride in anhydrous toluene and trimethylamine (Scheme 1).

The ligand was isolated as an air stable white solid at room temperature, and it was soluble in non-polar solvents such as dichloromethane and chloroform. Mass spectrometry and NMR, FTIR, and Raman spectroscopy analyses were used to characterize the isolated compound. The electrospray mass spectrum in acetonitrile showed the molecular ion peak at 291.062 *m*/*z*, which matched the calculated value for [(C_12_H_8_N_6_O_2_)Na]^+^ (Figure S4 in the Supplementary Materials). NMR spectroscopy confirmed the formation of the ligand. The ^1^H- and ^13^C-NMR spectrum chemical shifts were assigned with the aid of a heteronuclear single quantum coherence (HSQC) experiment. (Figures S1–S3 in Supplementary Materials). The solid-state IR spectra from a KBr pellet (Figure S5 in Supplementary Materials) showed bands attributable to C=O (1710 cm^−1^) [19] and C=C (1525 cm^−1^) [20]. Raman analysis showed 532 nm was the optimum wavelength for obtaining Raman dispersion information from the sample. There are four vibrational modes in the region of 250 to 1000 cm^−1^. These modes correspond to symmetric vibrations and are characteristic of the synthesized ligand.

### 2.2. Synthesis and Characterization of Coordination Polymers (CPs)

The coordination polymers (CPs) were obtained using simple methods of synthesis and purification. In the synthesis of **2**, it was necessary to dissolve the ligand in a 1:1 mixture of acetone and ethanol as the ligand was poorly soluble in pure acetone. The CP **2** was obtained as a non-hygroscopic blue solid and was stable in air at room temperature. In the case of the zinc complex (**4**), experimental and computational studies predict the formation of mononuclear complex, it was poorly soluble in common organic solvents, which facilitated purification. The solubility of **4** in DMSO and DMF allowed its characterization by NMR, which confirmed the formation of the ligand and the zinc CP. NMR spectra of **4** and the free ligand (**1**) showed similar signals. This is likely because complexation with the metal center does not affect the magnetic environments of the hydrogens in the ligand.

#### 2.2.1. Infrared Spectroscopy

A comparison of the vibrational bands of the ligand with those of the corresponding CPs showed that coordination of the ligand to the metal causes some bands to shift or disappear altogether (Table 1). In the free ligand, a band at 1710 cm^−1^ was observed and was attributed to the carbonyl group [21], and this (C=O) band was shifted in the spectrum of the CPs because the ligand was coordinated to the metal. In all cases, we observed a shift in the (C=O) band to lower wavenumbers. Those results indicate the lower rigidity of the (C=O) bonds in the CPs (Table 1).

#### 2.2.2. Thermal Analysis

The stages of decomposition, temperature ranges and decomposition products as well as the weight loss percentages of the CPs are shown in Table 2. The fragments listed in Table 2 are proposed based on the mass losses because detection of the actual fragments was not possible. We propose all the complexes studied here loose HCl and CO molecules. In addition, for **2** and **4**, we observed an initial loss that generated an unstable compound that rapidly degraded to a compound of greater stability, which was stable to approximately 100 °C, and then, we observed final losses of the corresponding metal residue. For **5**, three losses were observed, which are probably due to the formation of compounds of greater stability, so slow decays are observed in the mass percentage with respect to temperature. In contrast, five losses were observed for **3**, which are probably due to the formation of compounds of low stability, so fast decays are observed in the mass percentage with respect to temperature.

#### 2.2.3. X-Ray Diffraction Studies

**Figure 1:**
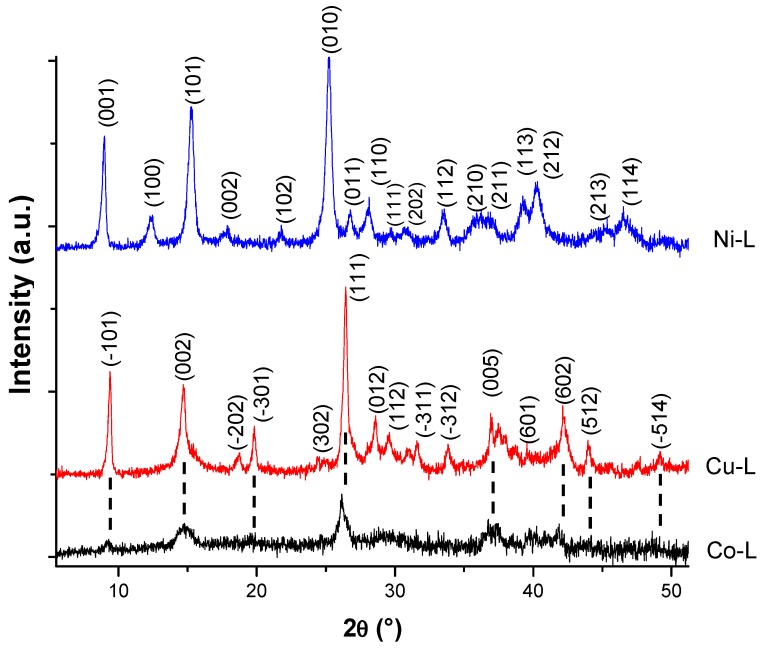
XRD patterns for Co, Cu and Ni CPs showing some of the *hkl* indices.

The XRD patterns for the Co, Cu and Ni-CPs are shown in Figure 1. The diffractograms were successfully indexed on a monoclinic unit cell using the DICVOL06 and Jana-2006 programs [22,23] with an absolute error of ±0.05° (2θ) in the calculations (Table 3). In all cases, an analysis of the patterns was performed using the Le Bail method [24]. The refinements were performed using a pseudo-Voigt profile function for the peak shape and a calculated background using a linear interpolation between a set of fixed points. Table 3 shows the refined unit cell parameters and the most likely space group estimated using Jana software [22].

In all cases, the formation of a single phase was confirmed through the X-ray analyses since no additional peaks from secondary phases have been identified after the Le Bail analysis. From Table 4, it is possible to imagine that the Co and Cu CPs. have similar crystal structures. However, the presence of Ni in the CPs breaks the two-fold rotoinversion axis observed in the Cu and Co CPs, and a two-fold screw axis in the [010] direction is present instead. The consequence of these changes in the symmetry is still under investigation due to difficulties in obtaining single crystals of adequate size. An approximation of the crystallite size (the size of the coherently diffracting domain) of the CPs was calculated using the Scherrer equation, D = (Kλ)/(βcosθ), using K as 0.89 [25]. The apparent crystallite sizes of the Co, Cu and Ni CPs are 17.0, 24.6 and 24.1 nm, respectively, which are consistent with the width and low intensity of the peaks. These peaks are more prominent in the case of the Co CP. A similar X-ray diffraction study was planned in the case of the Zn CP; however, it was not possible to find a solution that could explain the complete diffractogram (not shown), which suggests fast degradation of the CP even during the measurement.

#### 2.2.4. Molecular Modeling

**Figure 2:**
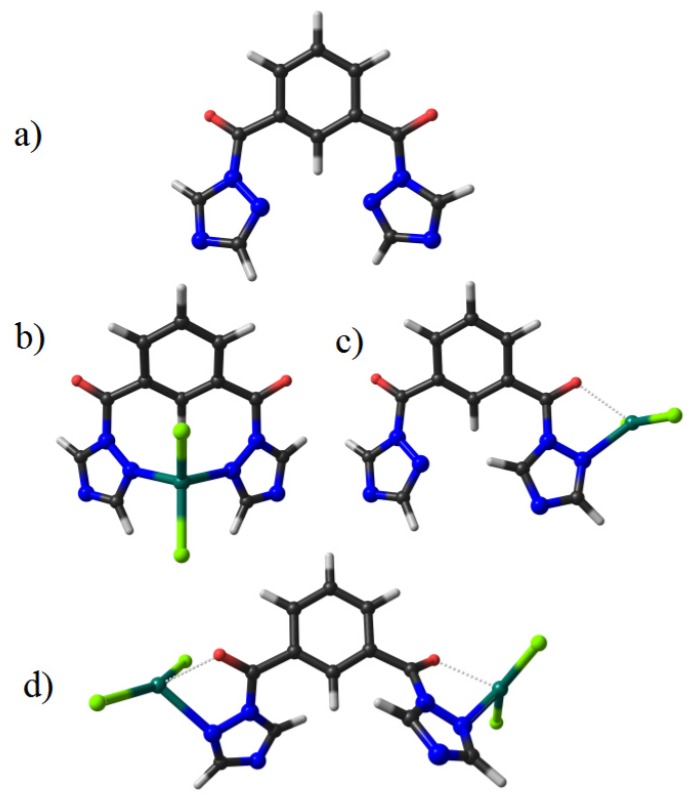
Representative structures for both ligand and metallic complexes. (**a**) Free ligand (**1**); (**b**) The metal is coordinated only via N,N of the triazolyl ligand; (**c**) The metal is coordinated via a C=O and a N from the triazolyl ligand moiety; (**d**) Two metals are coordinated via the C=O and N moieties of the triazolyl ligand. Calculations were done by using DFT TZ2P/BP86-D3 level of theory.

In order to evaluate probable structures for all the complexes, molecular modeling calculations were performed preliminary by semi-empirical methods (PM6), and followed by DFT calculations. Owing to the different coordinating sites provided by ligand **1**, different coordination modes were studied. The results are shown in Figure 2. Vibrational frequencies were calculated to determine the global minimum in the surface potential, owing to the absence of imaginary frequencies.

**Figure 3:**
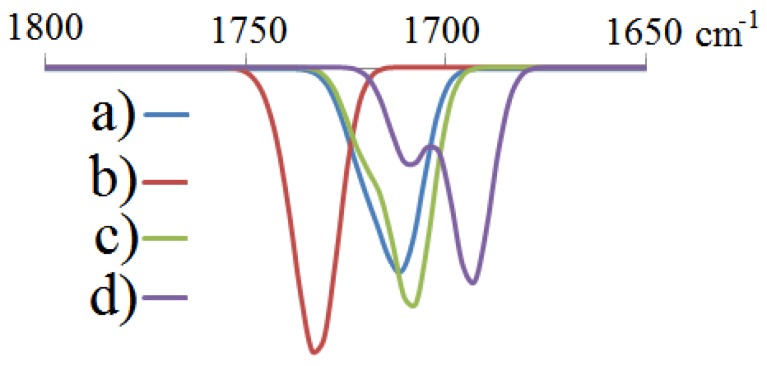
Simulated infrared spectra of the possible complexes. The letters (**a**–**d**) corresponds to the molecules in Figure 2, with M = Zn.

Because we observed shifts in the C=O band to lower wavenumbers (relative to the free ligand) in the experimental IR experimental spectra, we decided to computationally simulate the infrared spectra for the possible complexes and study the shift of the C=O band. The results are presented in Figure 3.

In general, the simulated IR patterns corresponded to those obtained experimentally for all metal complexes. In the computational study, we observed that the (C=O) band in **4** is at higher energy when the metal is coordinated only via the *N*,*N* from the triazoly ligand (Figure 2b). This result contradicts what was observed in the experimental IR spectra.

However, if the metal is coordinated via the C=O and one nitrogen of the triazole moiety (Figure 2c), a split carbonyl signal is produced since the free C=O vibration is different than the vibration for C=O coordinated to the metal (C=O–M). Finally, if the metals are symmetrically coordinated to the ligand via the C=O and N moieties (Figure 2d), the bands shift to lower wavenumbers as was found in the experimental IR spectrum.

**Figure 4:**
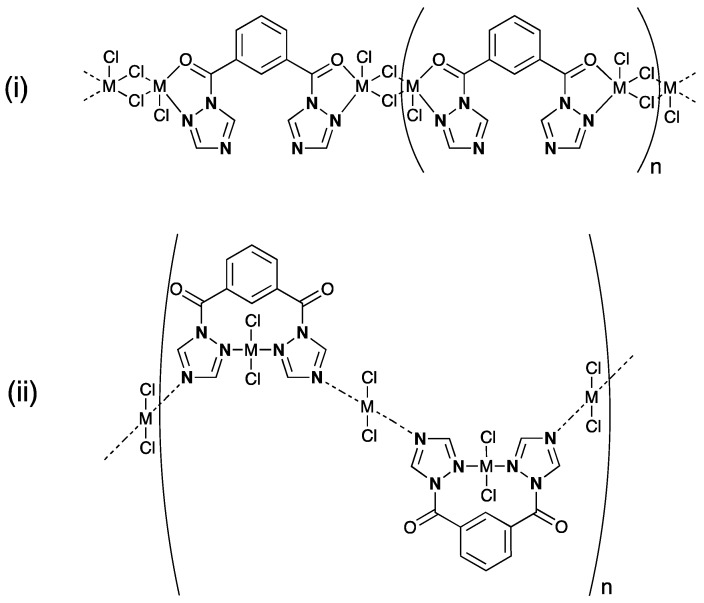
Probable structure for coordination polymers (**i**) **2** and **5**; (**ii**) for **3**.

The favored coordination modes provided by structures Figure 2b,c, were evaluated for Co(II), Ni(II), Cu(II) and Zn(II) at the DFT TZ2P/BP86-D3 level of theory. Interestingly, for Co(II) and Ni(II), the more stable structure is given by the coordination mode involving a C=O and a N from the triazolyl ligand moiety (Figure 2b and Figure 4i). In contrast, for Cu(II) and Zn(II), the metal is coordinated preferably only via N,N of the triazolyl ligand (Figure 3c and Figure 4ii).

Although mononuclear, binuclear and coordination polymers have been obtained using related bis(azolyl) ligands [26,27,28,29,30], in our case, the isolation of non-soluble compounds indicates the probable formation of a polymeric species for **2**, **3** and **5**. This assumption is supported by the results from both experimental and computational studies, and it was possible to propose structures of metal complexes (coordination polymers) as shown in Figure 4. Where for Co(II) and Ni(II), polymeric arrangement polymer (i) from Figure 4 is expected, and polymer (ii) is anticipated for Cu(II).

On the other hand, the elemental analysis results showed a (metal: ligand) of 2:1 ratio for **2**, **3** and **5**, corresponding to **L**(MCl_2_)_2_. Finally for the zinc complex **4**, the metal:ligand ratio found was 1:1 and its infrared spectrum showed no effect on the intensity of the C=O band. Based on the observed is proposed for **4** a mononuclear structure, where the metal is coordinated preferably only via *N*,*N* of the triazolyl ligand.

#### 2.2.5. ε-Caprolactone Polymerization

Other metal complexes have been studied as catalysts for CL polymerization [1,3,6,11,31,32]. However, some of them require special handling techniques such as high vacuum conditions and all-glass apparatus, which makes them difficult to use in a practical sense. Recently, we have been interested in catalysts for ROP that can be easily synthesized [12]. The behaviors of CPs **2**–**5** as initiators for polymerization of CL were tested. The solvent-free polymerization of ε-caprolactone was conducted at 110 °C for 72 h using a [monomer]:[CPs] molar ratio of 490:1. This ratio was selected because it represents the minimum catalyst loading relative to the monomer. Initiator **5** did not produce the desired PCL under the conditions studied. We think that this result may be due to its low solubility in the reaction medium or because the active species was not generated [5,12]. The polymers of PCL were obtained as white powders and were characterized by DSC, TGA, FTIR and ^1^H-NMR spectroscopy (Figures S20–S27 in Supplementary Materials). Table 4 shows the yield and DSC and TGA characterization data of the obtained PCL.

The results showed that the CPs and **4** were active in the ring opening polymerization of ε-caprolactone, and the yields were 50–57%. Longer reaction times did not increase the percent conversion. The activities of the CPs and **4** seem to be governed by their solubility in the reaction medium. The products all have melting points of 59 °C (Figures S22–S27 in the Supplementary Materials), which is characteristic of PCL [3], and high crystallinities (63–68%). The crystallinities were higher than that of commercial PCL (31% according to manufacturer’s report), which may be beneficial for applications in films. It has been reported that changes in the orientation and crystallization of the polymers can improve their barrier properties [32]. The obtained polymers showed crystallinity values similar to those of the PCL produced using a zinc catalyst or the chromium complexes we have reported [12,13].

Analyzing the results of TGA (Figures S28–S30 in the Supplementary Materials), the materials show decomposition temperatures above 400 °C, which suggest they are stable at high temperatures. The decomposition temperature of the PCL obtained when using **4** as the initiator was higher than that of the PCL produced using zinc complexes derived from coumarin, salen and Schiff base ligands [12,32,33]. The TGA and DSC results from the PCL product showed that these materials have potential applications in the design of biodegradable packaging.

## 3. Materials and Methods

### 3.1. General Information

All manipulations were routinely performed in an inert atmosphere (nitrogen) using standard Schlenk-tube techniques. All reagent-grade solvents were dried, distilled, and stored under a nitrogen atmosphere. Commercial PCL (reference No. 440744) was purchased from Sigma-Aldrich (St. Louis, MO, USA). Elemental analyses (C, H and N) were carried out on a Thermo Scientific™ FLASH 2000 CHNS/O Analyzer (Thermo Fisher Scientific, Waltham, MA, USA). Fourier transform infrared (FTIR) spectra were recorded on a Thermo Nicolet NEXUS FTIR spectrophotometer using KBr pellets or on a Thermo Scientific Nicolet 380 spectrophotometer. Nuclear magnetic resonance (NMR) spectra were recorded on a Bruker 400 spectrometer. Chemical shifts are reported in ppm relative to a SiMe_4_ (^1^H) internal standard. The mass spectra of the new ligand was obtained on a Micromass Quattro Q-TOF LC/. Melting points were determined on a Mel-Temp^®^ 1101D apparatus in open capillary tubes, and they are uncorrected. Thermogravimetric analyses (TGA) of the complexes and polymers (PCL) were obtained on a NETZSCH STA 409 PC/PG from 8 to 10 mg of the complexes in nitrogen media. The samples were subjected to dynamic heating over a temperature range of 30–700 °C with a heating rate of 10 °C min^−1^. The TG curves were analyzed to determine the percentage of mass lost as a function of temperature. Raman spectroscopy was performed in an RIBA Yovin-Ivon spectrometer using different laser wavelengths (532, 638, and 786 nm).

The molecular modeling calculations were performed by semi-empirical methods (PM6) with MOPAC2012 software, version 15.152 W [34,35], and Gabedit, version 2.3.8 [36]. Density Functional Theory (DFT) calculations, were done by using the ADF2016 package [37], with the dispersion corrected BP86 GGA exchange-correlation functional (BP86-D3) [38] in conjunction to all electron TZ2P basis set [39].

After synthesis, phase analysis was performed by X-ray diffraction (XRD) at room temperature using a Miniflex-Rigaku X-ray diffractometer working in Bragg-Brentano geometry with Cu-Kα_1,2_ wavelengths (1.54051 and 1.54433 Å). The diffractometer was operated over the angular range of 2θ = 10°–70° using a step size of 0.02° (2θ) and an acquisition time of 4 s per step.

Differential scanning calorimetry (DSC) analysis of the PCL was performed with a TA Instruments DSC Q200 instrument in a nitrogen atmosphere (50 mL min^−1^). An 8–10 mg sample was heated from 30 to 150 °C, cooled from 150 to −90 °C and heated from −90 to 90 °C at a heating rate of 5 °C min^−1^. The crystallinity was determined using the formula:

$$X_{c}=\frac{\Delta H_{exp}}{\Delta H_{u}^{0}}$$

where $\Delta H_{exp}$ is the area of the melting peak and $\Delta H_{u}^{0}$ is the melting enthalpy of 100% crystalline PCL (136.08 J g^−1^) [40].

### 3.2. Synthesis of 1,3-Phenylenebis(1,2,4-triazole-1-yl)methanone *(**1**)*

To a Schlenk tube equipped with a reflux condenser was added isophthaloyl dichloride (2.46 mmol; 499.5 mg), 1*H*-1,2,4-triazole (4.98 mmol; 345.0 mg), triethylamine (0.8 mL) and toluene (20 mL), and the mixture was refluxed for 36 h. The hot mixture was filtered, and the filtrate was evaporated to dryness. The solid residue was treated with water (25 mL) and extracted with dichloromethane (2 × 30 mL). The organic layer was separated and dried with magnesium sulfate, and the solvent was evaporated to dryness. The resulting white solid was washed with n-pentane and dried at 80 °C for 6 h. Yield: 355.0 mg (54%). M.p.: 322–323 °C. IR (KBr) ν/cm^−1^: 3120, 2925, 2809, 2625, 1710, 1525, 1377, 1272, 1121, 955, 723, 665, and 518. Atom numbering for **1** is as follows: 
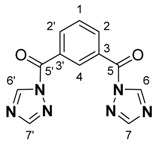
 ^1^H-NMR (400 MHz, DMSO-*d_6_*): δ 9.48 (s, 2H), 8.75 (t, 1H, *J* 1.7), 8.42 (dd, 4H, *J* 8.1 and 1.8 Hz), and 7.85 (t, 1H, *J* 7.9 Hz). ^13^C {^1^H}: δ 164.36 (C5, C5’), 154.22 (C7, C7’), 147.59 (C2, C2’), 136.86 (C6, C6’), 134.38 (C4), 131.25 (C3, C3’), and 129.32 (C1) Anal. calcd. for C_12_H_8_N_6_O_2_: C, 53.73; H, 3.01; and N, 31.33; found: C, 53.57; H, 3.04; and N, 29.65%. MS (FTMS + IT) *m*/*z*, calcd. for [M + Na]^+^: 291.062; found: 291.062.

### 3.3. Synthesis of Catena-Poly[chlorocobalt-di-μ-chloro-cobalt-μ-[1,3-Phenylenebis(1,2,3-triazole-1-yl)-methanone-O:N,O’:N’]] *(**2**)*

A solution of **1** (0.38 mmol; 101 mg) in a 1:1mixture of acetone:ethanol (4 mL) was added to a solution of cobalt(II) chloride (0.38 mmol; 49.5 mg) in acetone (5 mL). The reaction mixture was stirred at room temperature (rt) for 1 h. The resulting light blue solid was filtered off, washed with acetone and ethanol, and dried at 80 °C for 12 h. Yield: 97.0 mg (65%). M.p.: >400 °C (decomposition); IR (KBr) ν/cm^−1^: 3126, 3017, 2912, 2827, 2361, 1700, 1521, 1422, 1312, 1064, 884, 618, and 419. Anal. calcd. for C_12_H_8_Cl_4_Co_2_N_6_O_2_: C, 27.28; H, 1.52; and N, 15.91; found: C, 27.08; H, 1.51; and N, 15.20%.

### 3.4. Synthesis of Catena-Poly[chlorocopper-di-μ-chloro-copper-μ-[1,3-Phenylenebis(1,2,3-triazole-1-yl)-methanone-O:N,O’:N’]] *(**3**)*

A solution of **1** (0.74 mmol; 198.0 mg) in acetone (3 mL) was added to a solution of copper(II) chloride (0.75 mmol; 100.7 mg) in acetone (3 mL). The reaction mixture was stirred for 5 h at rt. The resulting blue solid was filtered off; washed with acetone, dichloromethane and diethyl ether; and dried at 80 °C for 12 h. Yield: 146.0 mg (49%). M.p.: 340 °C (decomposition). IR (KBr) ν/cm^−1^: 3448, 3135, 3024, 2926, 1708, 1519, 1427, 1373, 1215, 1079, 955, 876, 717, and 620. Anal. calcd. for C_12_H_8_Cl_4_Cu_2_N_6_O_2_: C, 26.81; H, 1.49; and N, 15.64; found: C, 26.61; H, 1.50; and N, 15.67%.

### 3.5. Synthesis of Catena-Poly[chlorozinc-di-μ-chloro-zinc-μ-[1,3-Phenylenebis(1,2,3-triazole-1-yl)-methanone-O:N,O’:N’]] *(**4**)*

A solution of **1** (0.188 mmol; 50.5 mg) in acetone (2 mL) was added to a solution of zinc(II) chloride (0.19 mmol; 26.5 mg) in acetone (1 mL). The reaction mixture was stirred for 1 h at rt. The resulting white solid was filtered off, washed with acetone and ethanol, and dried under a vacuum. Yield: 31.0 mg (40%). M.p.: >400 °C (decomposition); IR (KBr) ν/cm^−1^: 3118, 1691, 1418, 1223, and 420. ^1^H NMR (400 MHz, DMSO-*d_6_*): δ 9.49 (s, 2H, 2,2’), 8.76 (t, 1H, *J* 1.7 Hz), 8.42 (dd, 4H, *J* 8.1 and 1.8 Hz), and 7.85 (t, 1H, *J* 7.9 Hz). Anal. calcd. for C_12_H_8_Cl_2_ZnN_6_O_2_: C, 35.60; H, 1.98; and N, 20.76; found: C, 35.15; H, 1.88; and N, 20.72%.

### 3.6. Synthesis of Catena-Poly[chloronickel-di-μ-chloro-nickel-μ-[1,3-Phenylenebis(1,2,3-triazole-1-yl)-methanone-O:N,O’:N’]] *(**5**)*

To a Schlenk tube equipped with a reflux condenser was added nickel(II) chloride (0.39 mmol; 49.8 mg) in methanol (12 mL) and the mixture was refluxed for 30 min. Then, **1** (0.60 mmol; 160.2 mg) was added, and the mixture was refluxed for an additional 72 h. The resulting light violet solid was filtered off, washed with tetrahydrofuran and diethyl ether, and dried at 80 °C for 12 h. Yield: 94.0 mg (63%). M.p.: >400 °C (decomposition). IR (KBr) ν/cm^−1^: 3134, 2907, 1630, 1524, 1427, 1310, 1132, 1067, and 309. Anal. calcd. for C_12_H_8_Cl_4_Ni_2_N_6_O_2_: C, 27.30; H, 1.52; and N, 15.93; found: C, 27.30; H, 1.34; and N, 15.89%.

### 3.7. Polymerization of ε-Caprolactone

The polymerization of *ε*-caprolactone was performed solvent-free in four reaction tubes using [monomer]:[initiator] ([M]/[CPs]) ratios of 490:1 with 0.0036 mmol of CP and 0.178 mmol of *ε*-caprolactone. The reactions were carry out at 110 °C for 72 h. After the reaction time, the mixtures were cooled to room temperature. The polymers were purified by dissolving the crude product in dichloromethane (1 mL), and cold ethanol (7 mL) was added to give a white precipitate. The precipitate was then isolated by centrifugation, washed with HCl (7 mL, 0.1 M) (to remove traces of the catalyst) and ethanol (7 mL), and dried under vacuum at 40 °C [12,40]. The polymers were characterized by DSC, TGA, NMR and FTIR spectroscopy. IR (KBr; cm^−1^): *ν* 2945(C-H), 1725(C=O ester), 1241, 1185 (C–O–C). ^1^H-NMR (400 MHz, CDCl_3_): *δ* 4.09 (t, 2H), 2.38 (t, 2H), 1.72 (m, 4H), 1.41 (m, 2H) ppm. These data suggest that the obtained polymers were linear and have terminal hydroxymethylene groups [11].

## 4. Conclusions

We synthesized and characterized new metal complexes prepared via the reaction of 1,3-phenylenebis(1,2,4-triazol-1-yl)methanone with CoCl_2_, CuCl_2_, ZnCl_2_ and NiCl_2_. Experimental and computational studies predict the formation of coordination polymers (CPs). The prepared cobalt, copper and zinc CPs were good initiators for the ring-opening polymerization of *ε*-caprolactone under solvent-free conditions and afforded polymers with high crystallinities (63–68%) and decomposition temperatures above 400 °C. The results of TGA and DSC of the PCL showed that these materials have potential applications in the design of biodegradable packaging.

## Figures and Tables

**Table 1:** Infrared spectral bands for ligand 1 and its CPs.

Compound	Wavenumber ν/cm^−1^				
	ν(C–H)	ν(C=O)	ν(N–N)	ν(C–N)	ν(M–N)
**1**	3121 s	1710 vs	1429 m	1217 m	--
**2**	3126 s	1700 m	1427 m	1211 m	419 w
**3**	3135 s	1708 vs	1422 m	1215 m	417 w
**4**	3118 s	1691 vs	1418 m	1223 w	420 w
**5**	3134 s	1629 m	1427 m	1132 m	309 m

w: weak; m: medium; s: strong; vs: very strong.

**Table 2:** Thermoanalytical results (TGA and DTG) for the CPs.

Compound (Formula)	TG Range/°C	DTG_max_/°C	*n*	Mass Loss	Total Mass Loss	Assignment	Metallic Residue
				Estimated (calcd.)/%			
**2** (C_12_H_8_Cl_2_CoN_6_O_2_)	25–319	226	1	29.46 (30.53)	78.95 (78.29)	loss of C_2_H_2_N_3_	CoCl
	319–500	395, 432, 444	3	30.59 (32.34)		loss of C_6_H_4_ + CO + HCl	
	500–695	631	1	18.84 (15.42)		loss of C_2_NH_3_	
**3** (C_12_H_8_Cl_2_CuN_6_O_2_)	25–216	190	1	13.83 (15.76)	87.07 (84.22)	loss of HCl + CO	Cu
	216–368	287, 312, 320	3	53.15 (52.06)		loss of C_9_H_7_N_3_O + HCl	
	368–697	598	1	20.09 (16.40)		loss of C_2_N_3_	
**4** (C_12_H_8_Cl_2_N_6_O_2_Zn)	21–221	211	1	26.49 (24.70)	88.52 (83.84)	loss of 2HCl + CO	Zn
	221-300	281	1	31.62 (35.64)		loss of C_2_HN_3_ + C_6_H_5_	
	300–436	403	1	14.77 (13.60)		loss of C_2_HN_2_O	
	436–495	476	1	15.64 (9.90)		loss of CN_2_	
**5** (C_12_H_8_Cl_2_NiN_6_O_2_)	37–411	291	1	34.24 (36.04)	71.95 (74.95)	loss of C_6_H_4_ + CO + HCl	NiCl
	411–545	345	1	22.52 (22.99)		loss of C_2_H_2_N_3_ + CO	
	545–695	111	1	14.22 (16.05)		loss of C_2_HN_3_	

TG: thermogravimetric analysis; DTG: derivative thermogravimetric; *n*: number of decomposition steps.

**Table 3:** Refined unit cell parameters obtained from the Le Bail method for CPs.

Complex	Co	Cu	Ni
*a* (Å)	14.236 (19)	14.171 (2)	7.180 (2)
*b* (Å)	3.629 (4)	3.6617 (5)	3.5372 (5)
*c* (Å)	12.250 (16)	12.232 (2)	9.950 (3)
*β* (°)	94.36 (9)	94.21 (2)	90.65 (2)
*V* (Å^3^)	631 (1)	633.0 (2)	252.71 (15)
Space Group	*P*2_1_/*m*	*P*2_1_/*m*	*P*2/*m*
Rp	2.76	4.56	3.88
wRp	3.62	5.97	5.04
GOF	1.02	1.19	1.13

**Table 4:** Yield and characterization of the obtained PCL by DCS and TGA.

Initiator	(%) Yield	Melting point ^a^ (°C)	Crystallization T (°C)	Crystallinity ^b^ (%)	Decomposition T (°C)
**2**	57	59.0	37.24	67	435
**3**	56	59.8	37.09	68	440
**4**	50	59.6	38.33	63	445

All the materials were synthesized with a [monomer]/[CPs] mass ratio of 490:1 at 110 °C for 72 h. [a] The value correspond to the first heating ramp. [b] Calculated using a 100% crystalline ε-PCL enthalpy of fusion equal to 136 J/g [3].

## Supplementary Materials

The following are available online.
