# Supplementary material for: Coordination Polymers Containing 1,3-Phenylenebis-((1H-1,2,4-triazol-1-yl)methanone) Ligand: Synthesis and ε-Caprolactone Polymerization Behavior
Source: Molecules. 2017 Oct 29;22(11):1860. doi: 10.3390/molecules22111860 (PMC6150338; doi:10.3390/molecules22111860)
Supplement: Supplementary file 1 [file molecules-22-01860-s001.pdf]

Supplementary Material

Coordination polymers containing 1,3-phenylenebis((1H-1,2,4-triazol-1-yl)methanone) ligand: Synthesis and ε-caprolactone polymerization behavior

Nestor J. Bello-Vieda[1], Ricardo A. Murcia[1], Alvaro Muñoz-Castro[2,3], Mario A. Macías[1], John J. Hurtado[1]*

[1] Department of Chemistry, Universidad de los Andes, Carrera 1 N° 18A-12, 111711, Bogotá, Colombia; nj.bello1211@uniandes.edu.co, ra.murcia@uniandes.edu.co, ma.maciasl@uniandes.edu.co, jj.hurtado@uniandes.edu.co.

[2] Grupo de Química Inorgánica y Materiales Moleculares, Universidad Autonoma de Chile, El Llano Subercaseaux 2801, Santiago, Chile. aemunozc@gmail.com

[3] Relativistic Molecular Physics (ReMoPh) Group, Universidad Andres Bello, Republica 275, Santiago, Chile

* Correspondence: jj.hurtado@uniandes.edu.co Tel.: +57-3394949 ext 3468

Contents

1. Characterization of 1,3-phenylene-bis(1,2,4-triazole-1-yl)methanone (**1**).

2. Characterization of coordination polymers

3. Characterization of ε-caprolactone

4. The Raman discussion

1. Characterization of 1,3-phenylene-bis(1,2,4-triazole-1-yl)methanone (1)

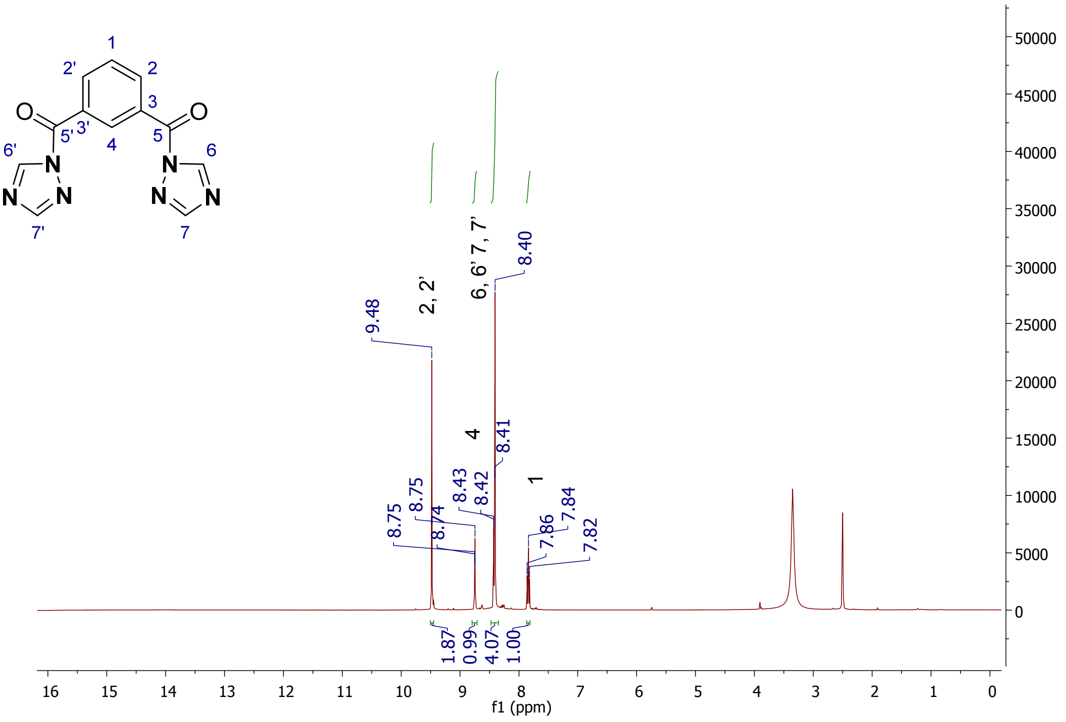

Figure S1. ^{1}H NMR spectrum of 1,3-phenylene-bis(1,2,4-triazole-1-yl)methanone (**1**) in $CDCl_3$

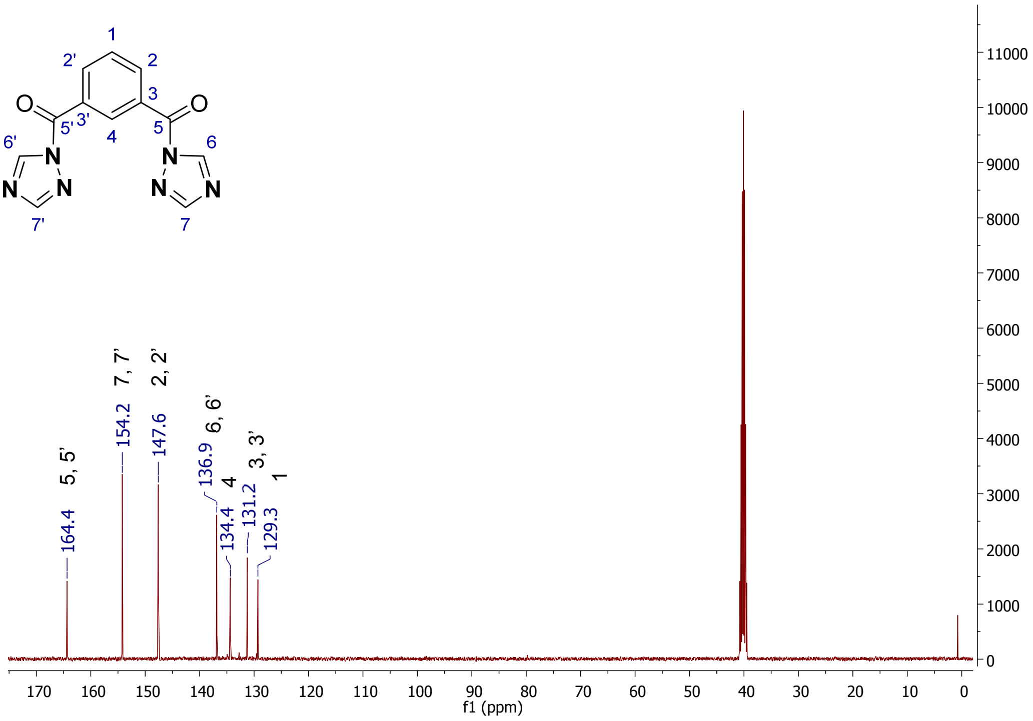

Figure S2. ^{13}C NMR spectrum of 1,3-phenylene-bis(1,2,4-triazole-1-yl)methanone (**1**) in $CDCl_3$

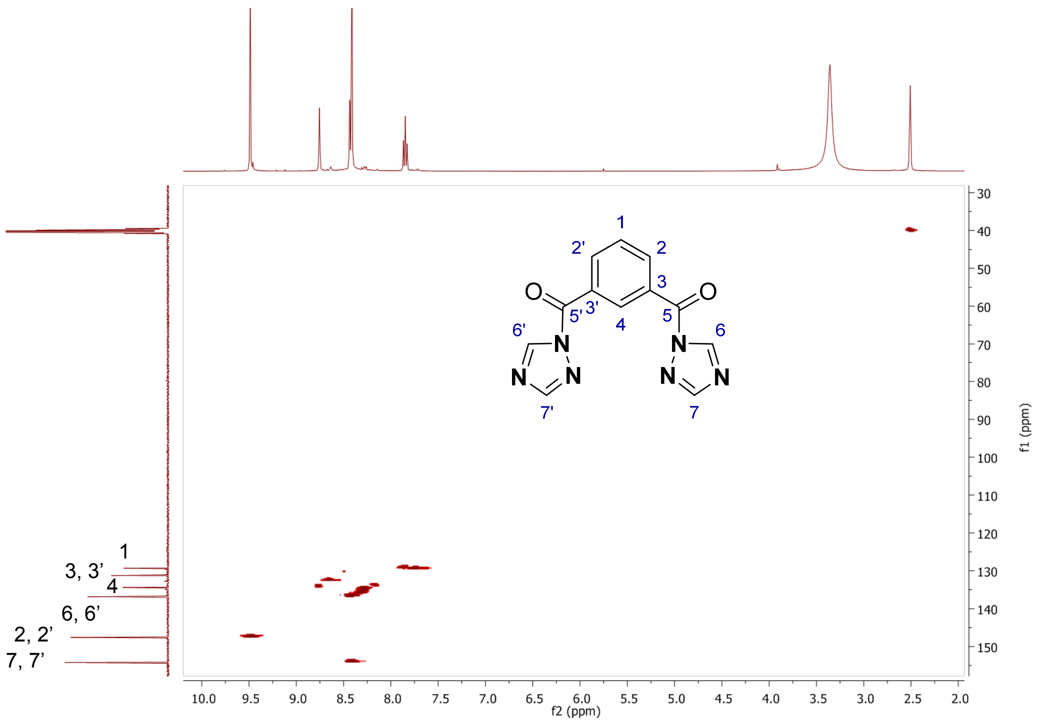

Figure S3. HSQC of 1,3-phenylene-bis(1,2,4-triazole-1-yl)methanone (**1**) in $CDCl_3$

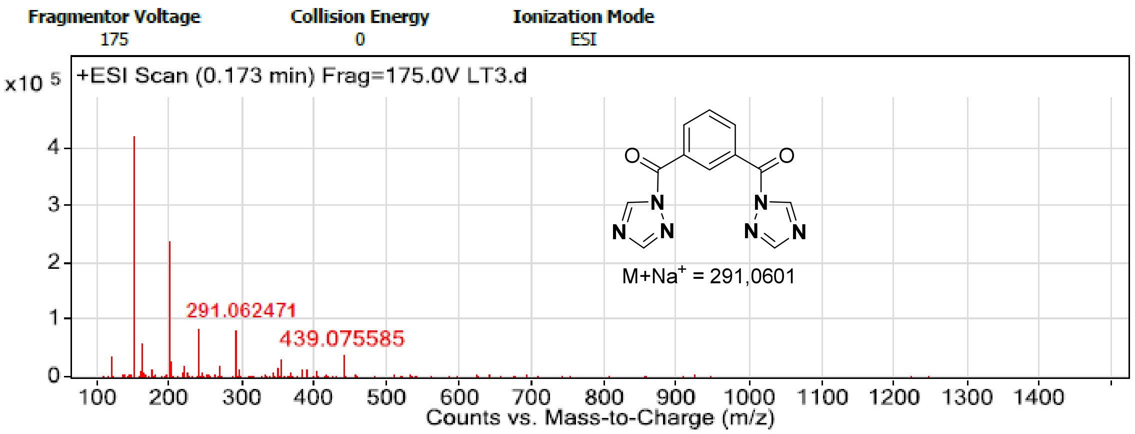

Figure S4. HRMS (ESI+) spectrum of (**1**) in acetonitrile

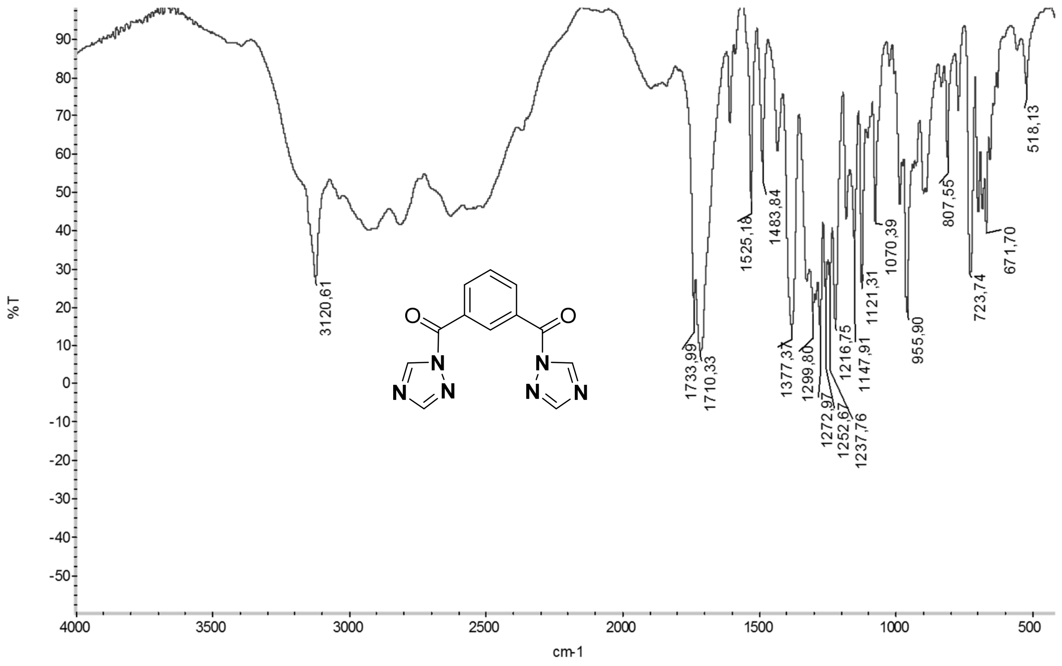

Figure S5. FT-IR (KBr) spectrum of 1,3-phenylene-bis(1,2,4-triazole-1-yl)methanone (**1**)

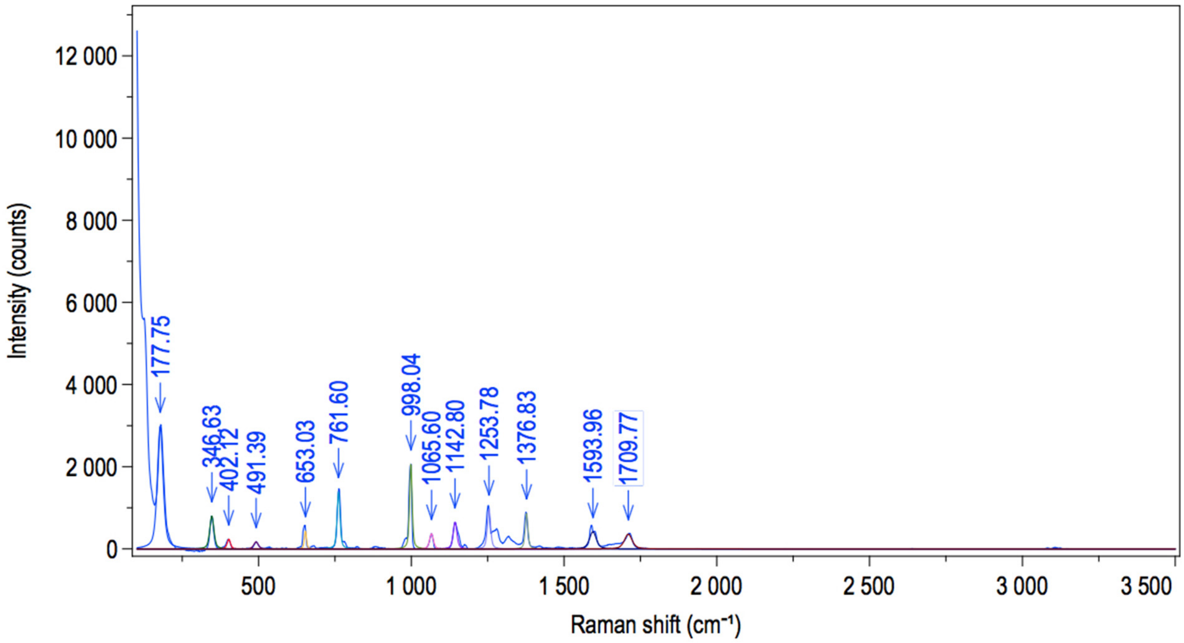

Figure S6. Raman spectrum of 1,3-phenylene-bis(1,2,4-triazole-1-yl)methanone ligand (**1**).

2. Characterization of coordination polymers

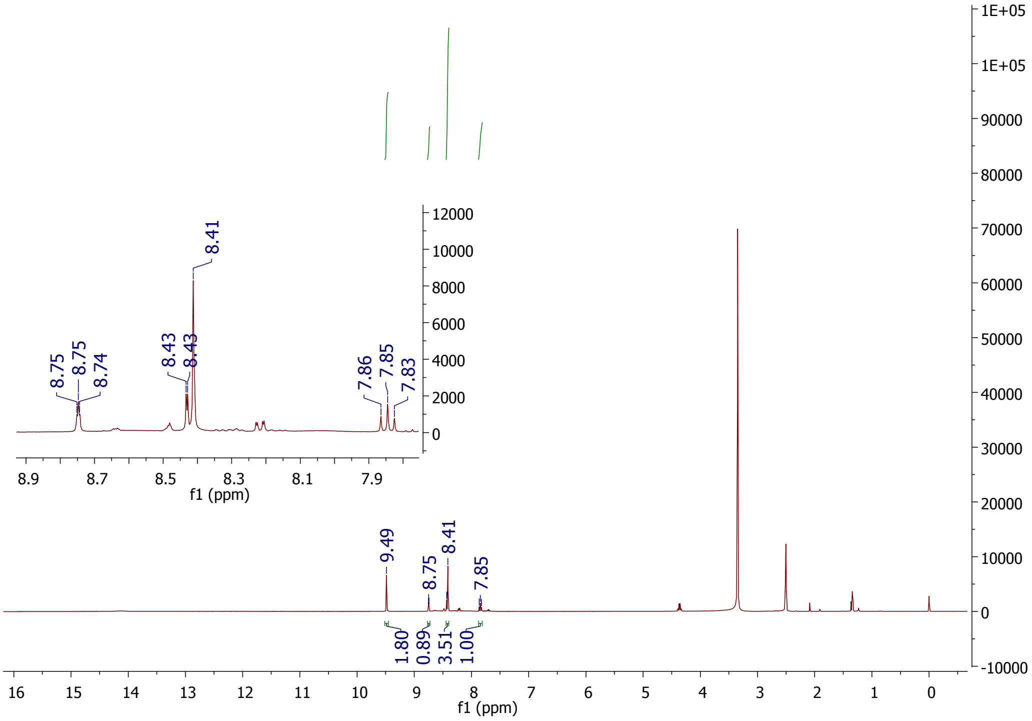

Figure S7. ¹H RMN spectrum of catena-poly[chlorozinc-di-μ-chloro-zinc-μ-[1,3-phenylenebis(1,2,3-triazole-1-yl)methanone-O:N,O':N']] (**4**) in $CDCl_3$

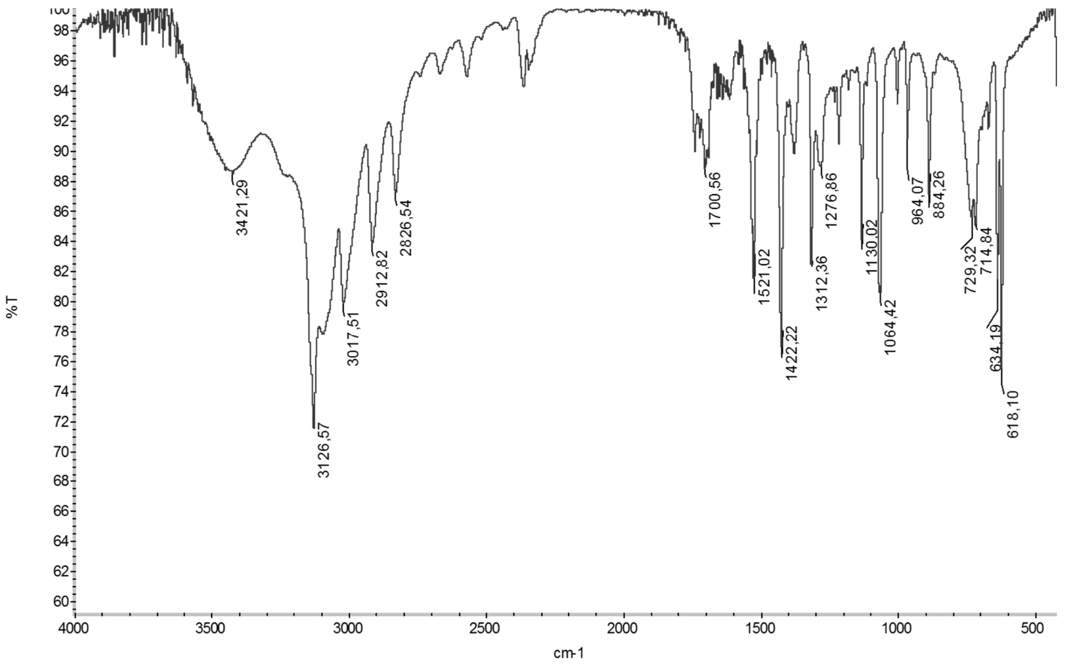

Figure S8. FT-IR (KBr) of catena-poly[chlorocobalt-di-μ-chloro-cobalt-μ-[1,3-phenylenebis(1,2,3-triazole-1-yl)methanone-O:N,O':N']] (**2**)

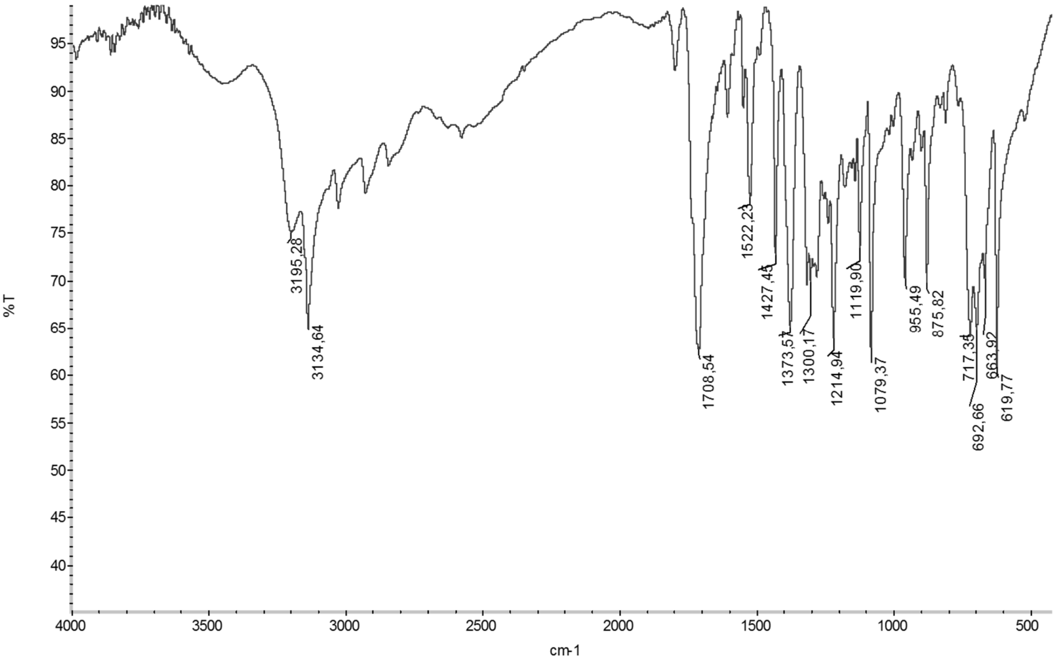

Figure S9. FT-IR (KBr) of catena-poly[chlorocopper-di-μ-chloro-copper-μ-[1,3-phenylenebis(1,2,3-triazole-1-yl)methanone-O:N,O':N']] (**3**)

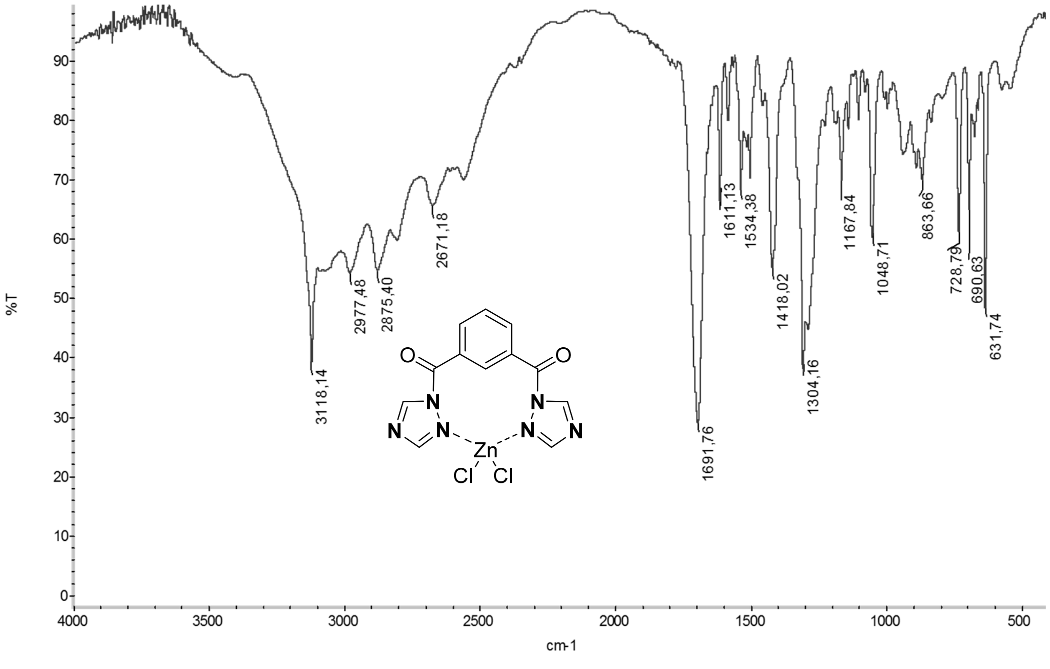

Figure S10. FT-IR (KBr) of catena-poly[chlorozinc-di-μ-chloro-zinc-μ-[1,3-phenylenebis(1,2,3-triazole-1-yl)methanone-O:N,O':N']] (**4**)

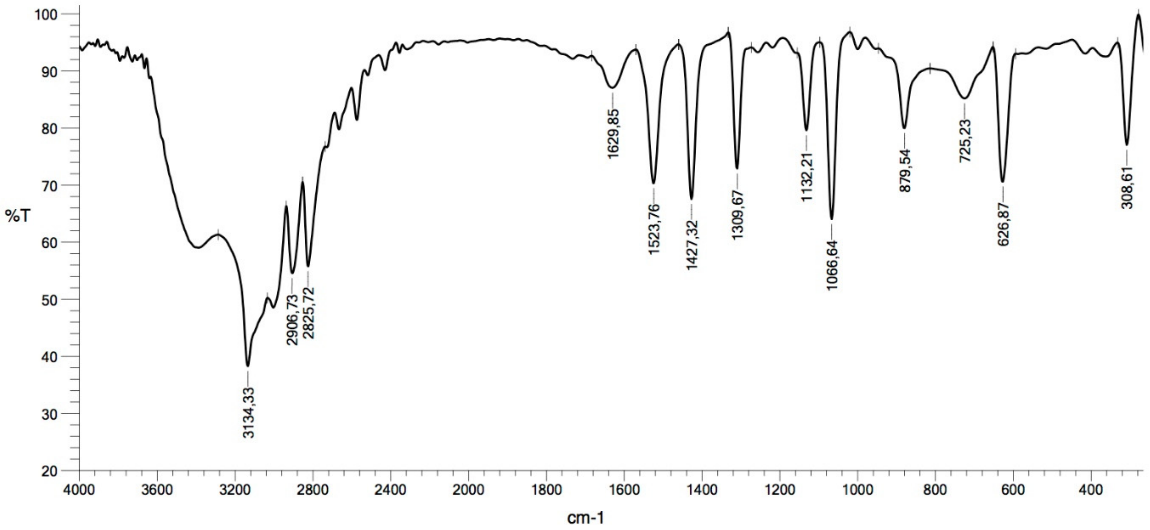

Figure S11. FT-IR (KBr) of catena-poly[chloronickel-di-μ-chloro-nickel-μ-[1,3-phenylenebis(1,2,3-triazole-1-yl)methanone-O:N,O':N']] (**5**)

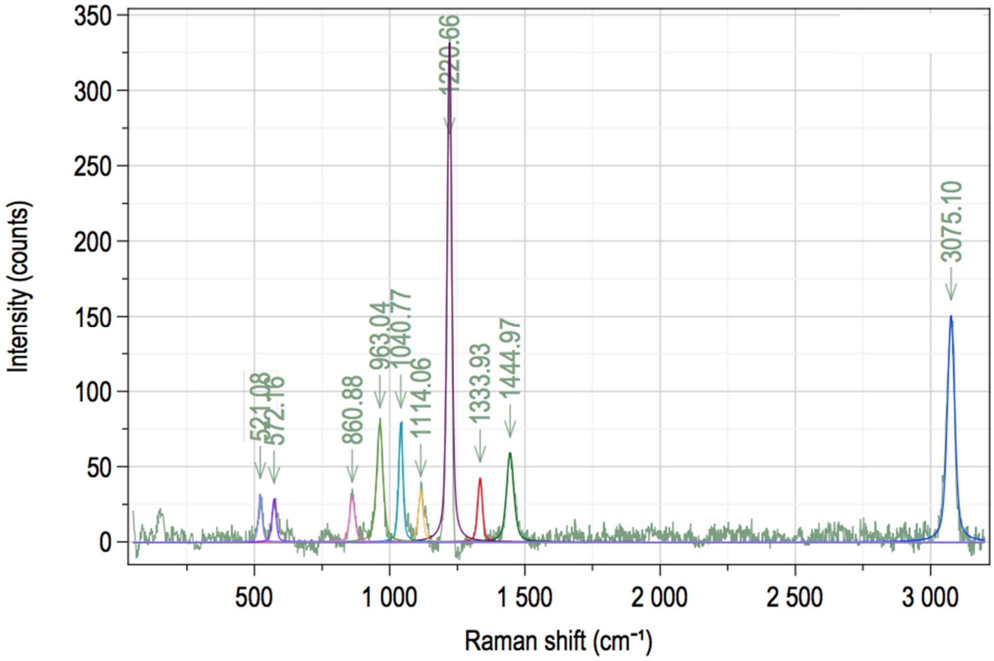

Figure S12. Raman spectrum of (**2**).

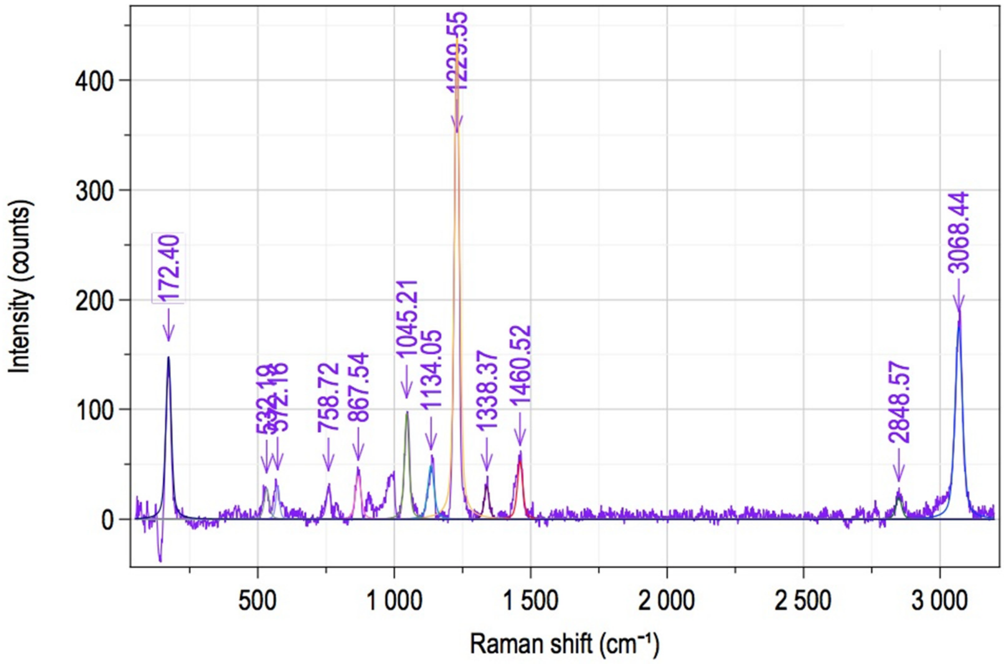

Figure S13. Raman spectrum of (**3**).

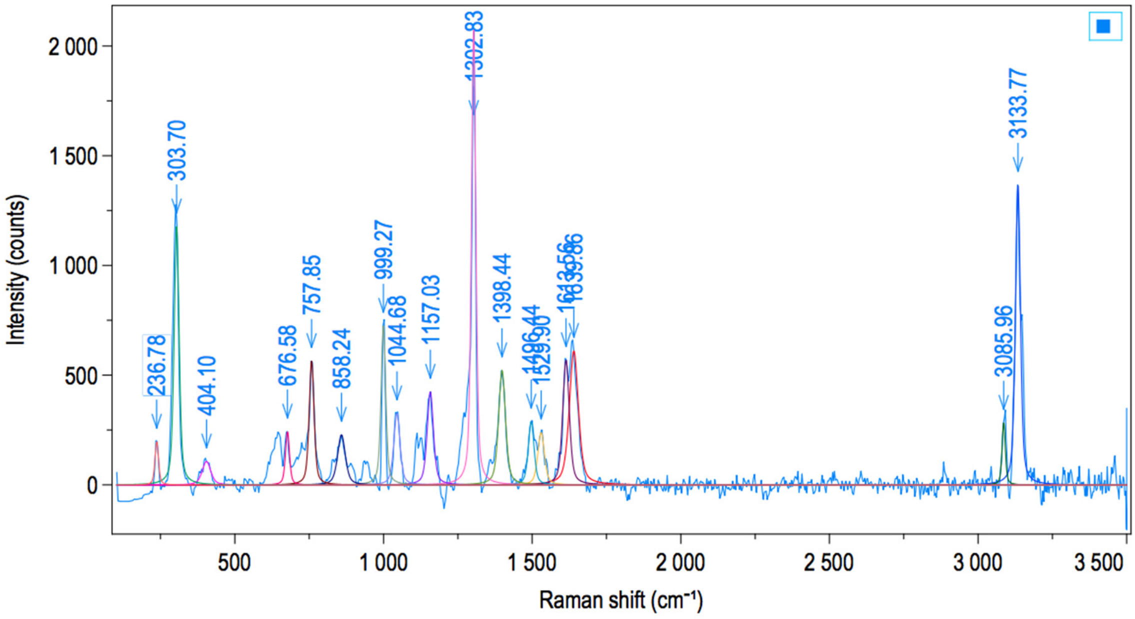

Figure S14. Raman spectrum of (**4**).

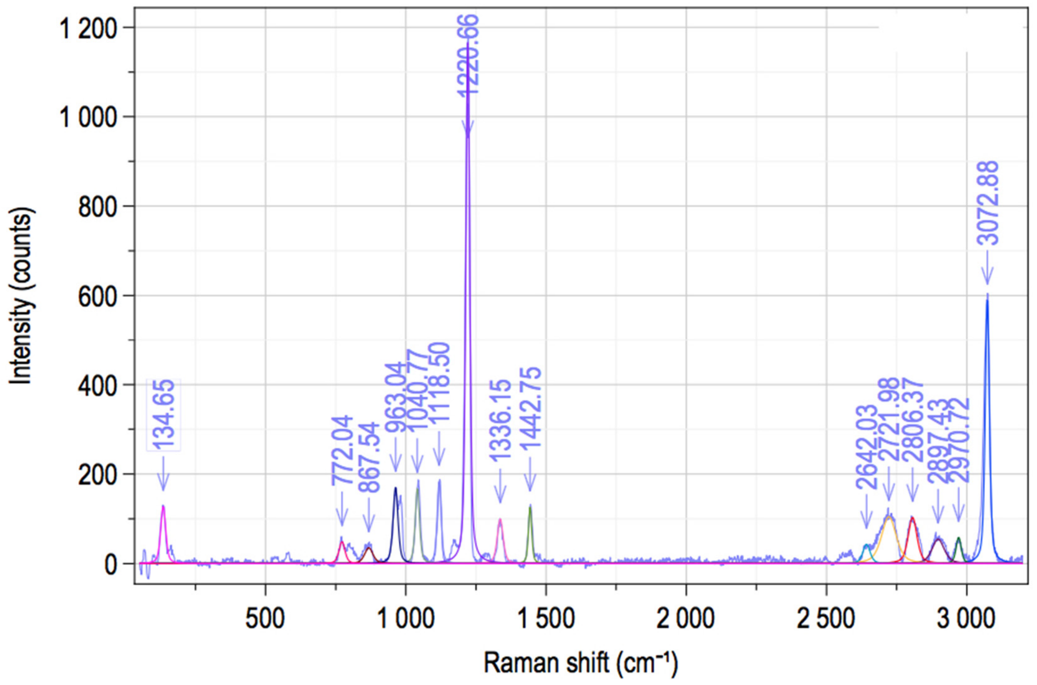

Figure S15. Raman spectrum of (**5**).

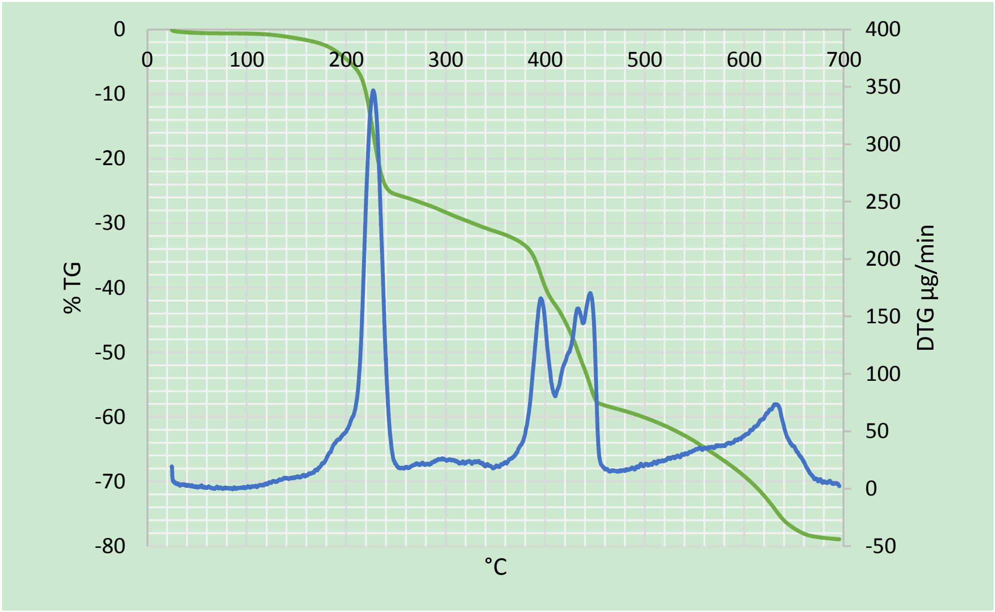

Figure S16. Thermogravimetric (TG) analysis and derivative thermogravimetric (DTG) of (**2**) in nitrogen atmosphere.

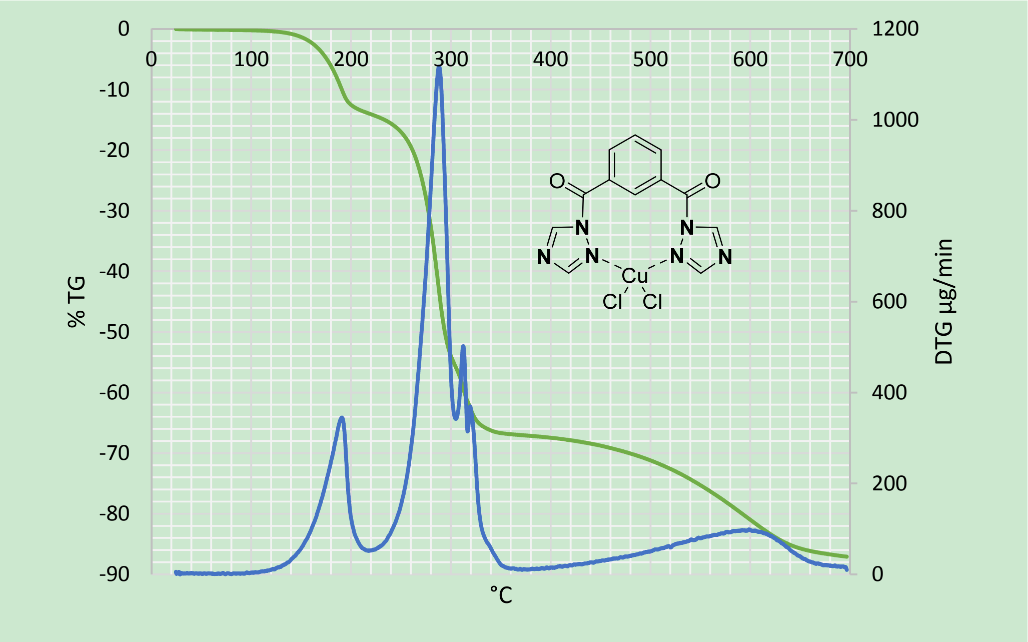

Figure S17. Thermogravimetric (TG) analysis and derivative thermogravimetric (DTG) of (**3**) in nitrogen atmosphere.

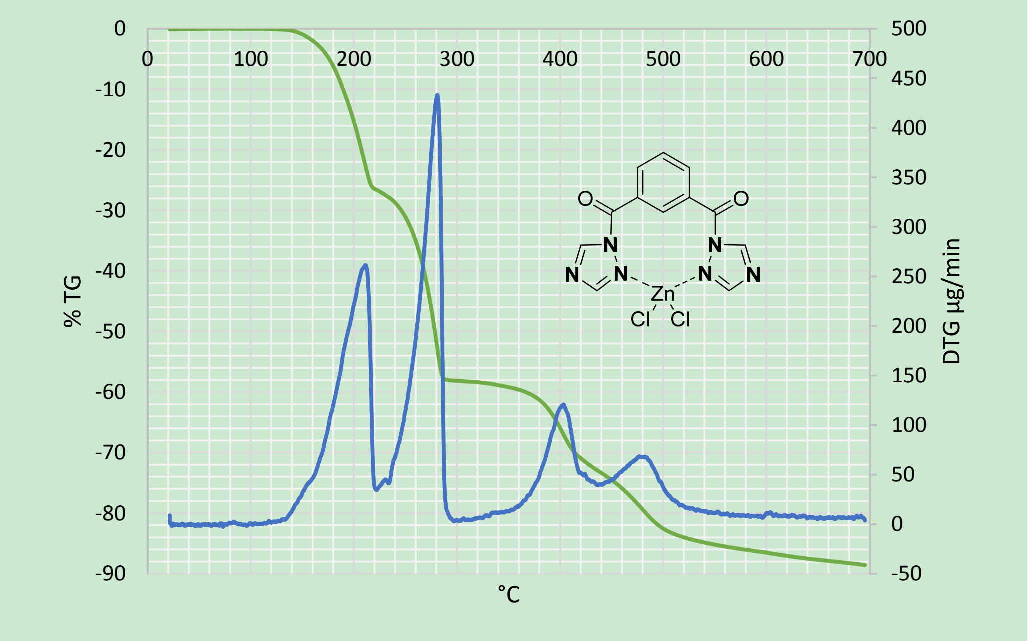

Figure S18. Thermogravimetric (TG) analysis and derivative thermogravimetric (DTG) of (**4**) in nitrogen atmosphere.

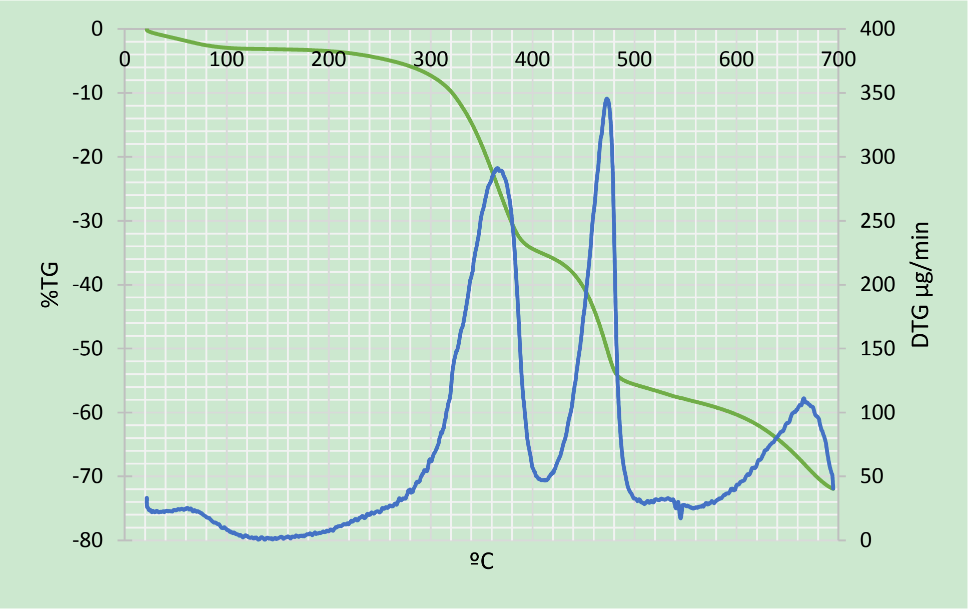

Figure S19. Thermogravimetric (TG) analysis and derivative thermogravimetric (DTG) of (**5**) in nitrogen atmosphere.

3. Characterization of PCL obtained

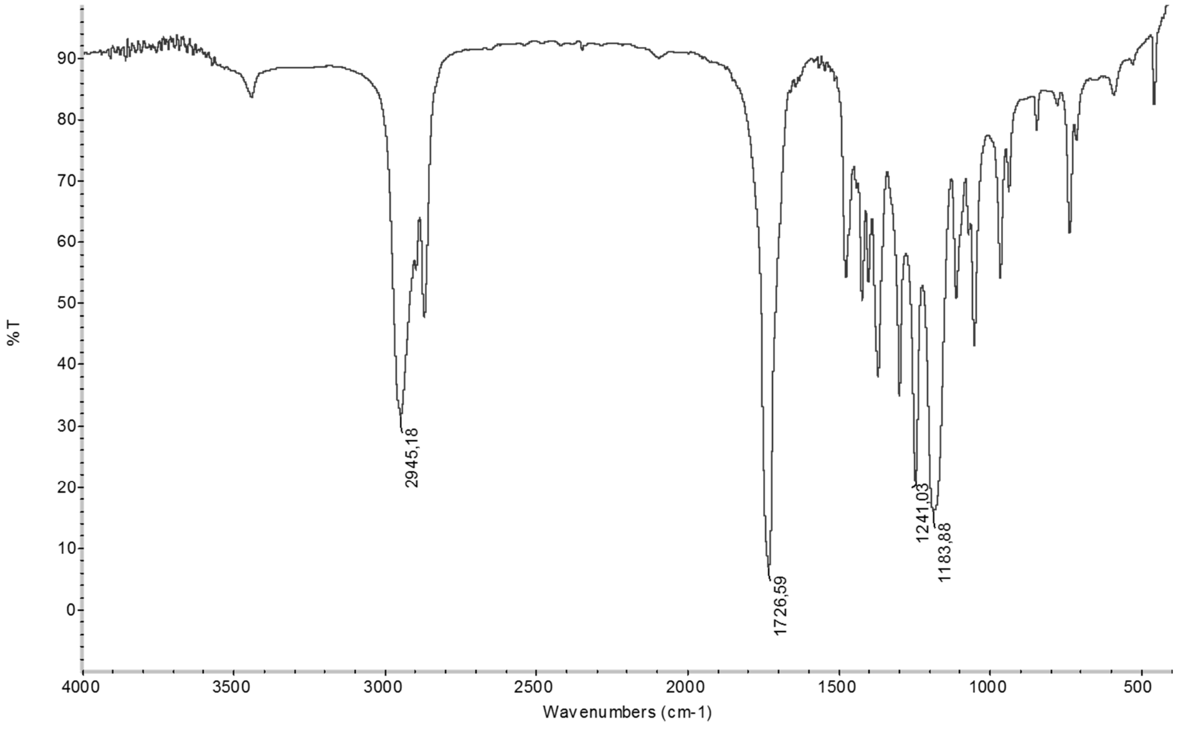

Figure S20. FT-IR (KBr) of polymer obtained with initiator **4**.

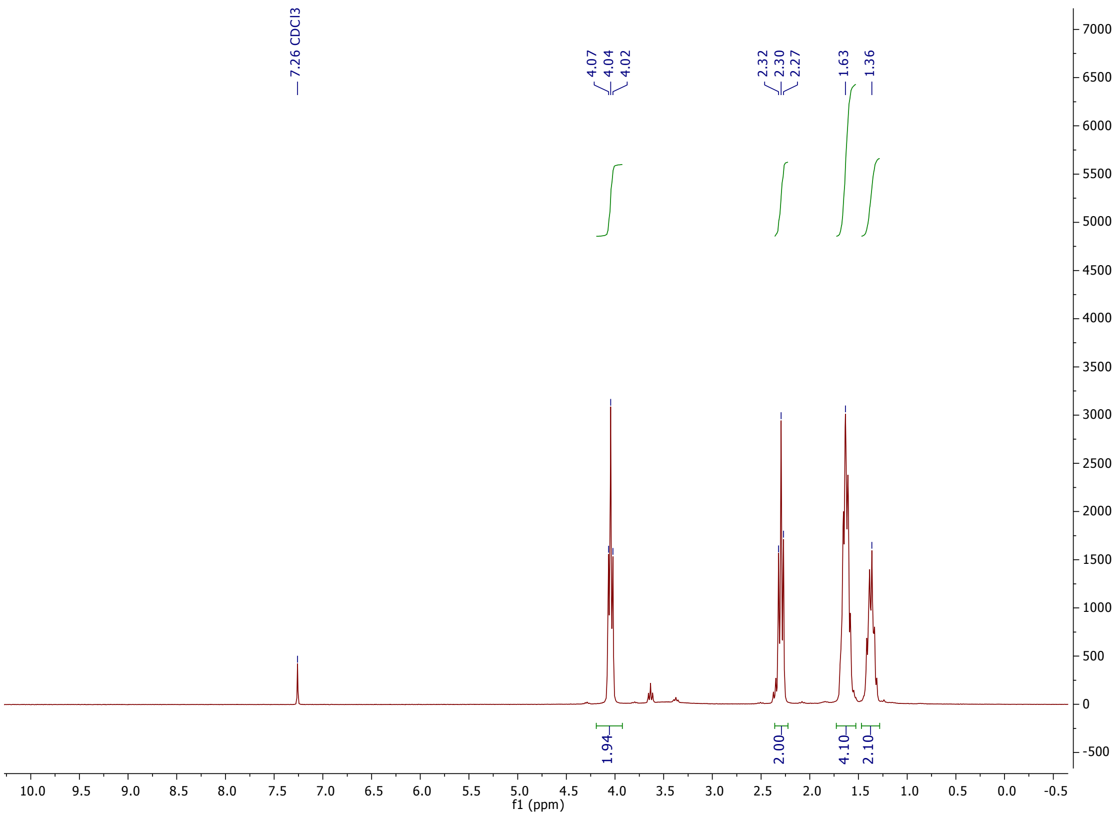

Figure S21. H[1] NMR spectrum of polymer obtained with initiator **4**.

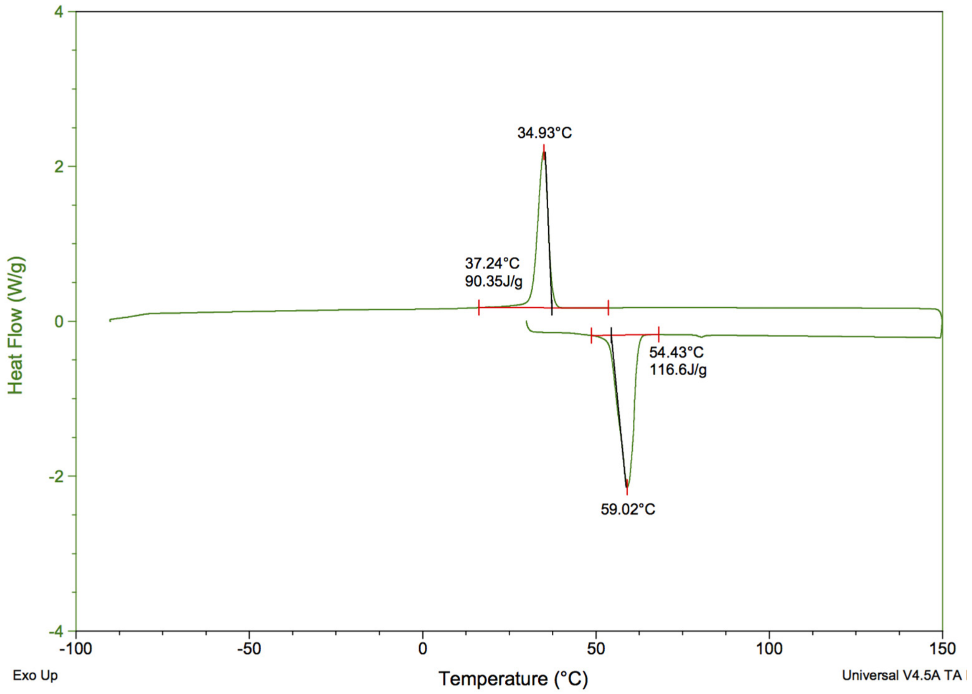

Figure S22. DSC of polymer obtained with initiator **2**. First heating and cooling ramp.

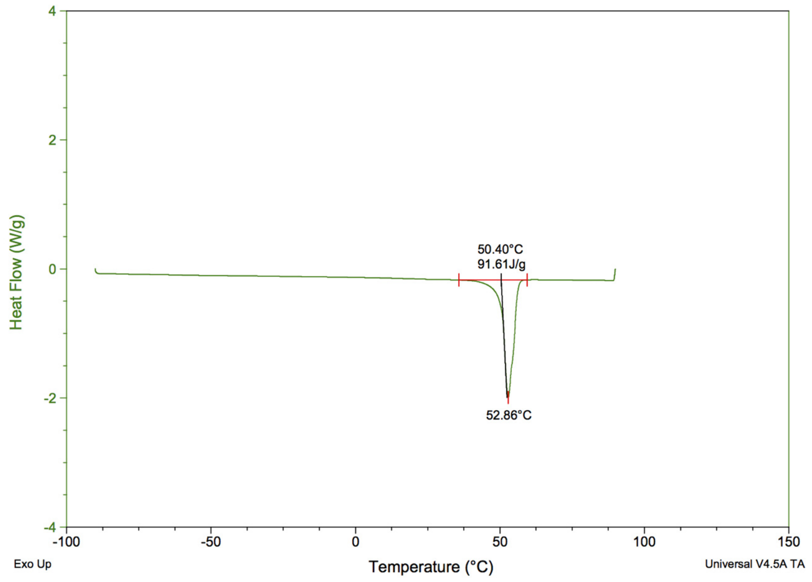

Figure S23. DSC of polymer obtained with initiator **2**. Second heating ramp

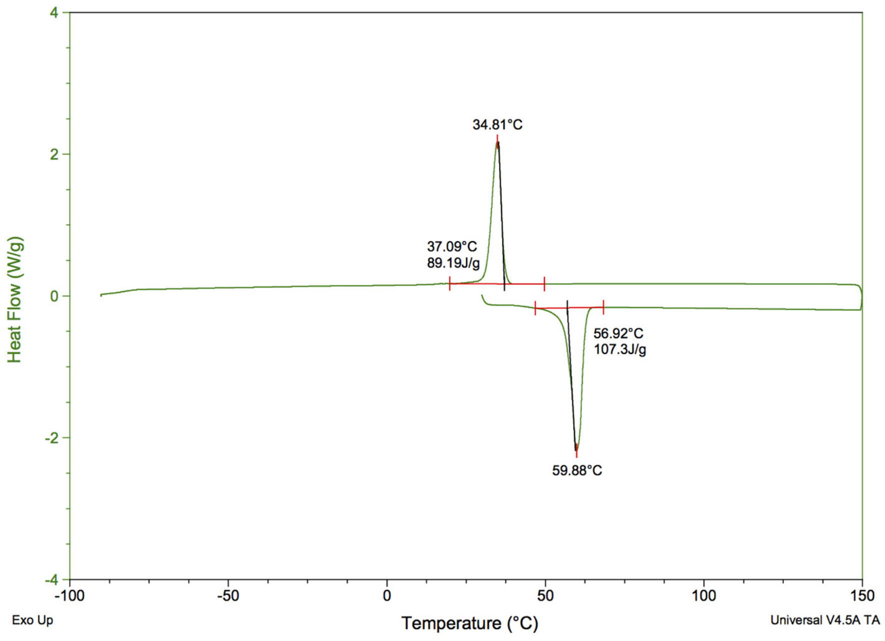

Figure S24. DSC of polymer obtained with initiator **3**. First heating and cooling ramp.

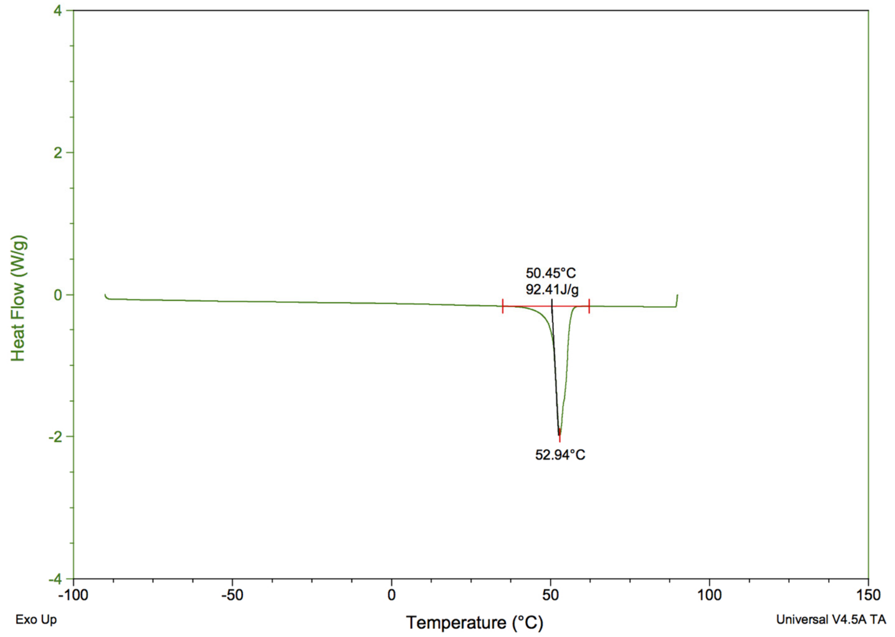

Figure S25. DSC of polymer obtained with initiator **3**. Second heating ramp

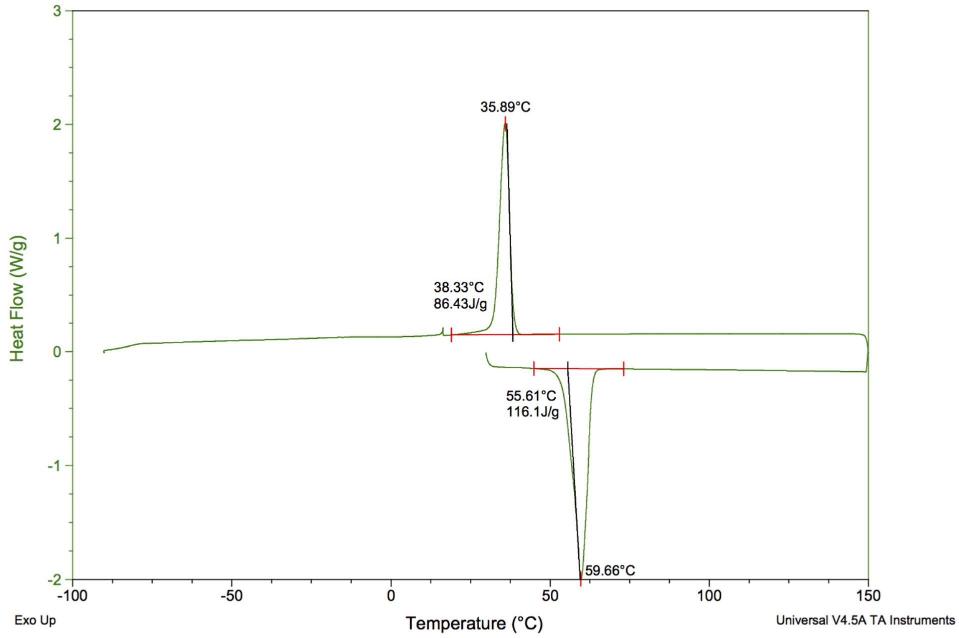

Figure S26. DSC of polymer obtained with initiator **4**. First heating and cooling ramp

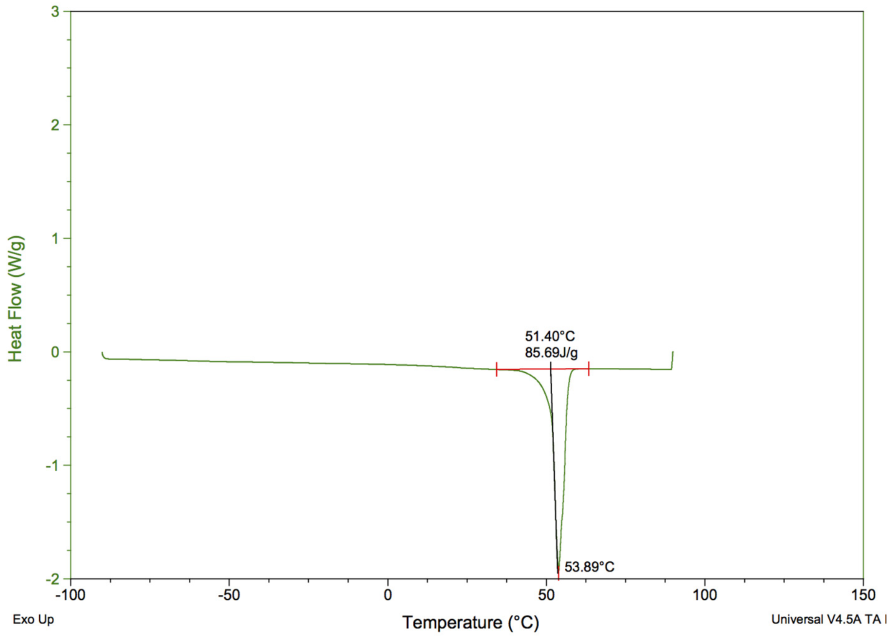

Figure S27. DSC of polymer obtained with initiator **4**. Second heating ramp

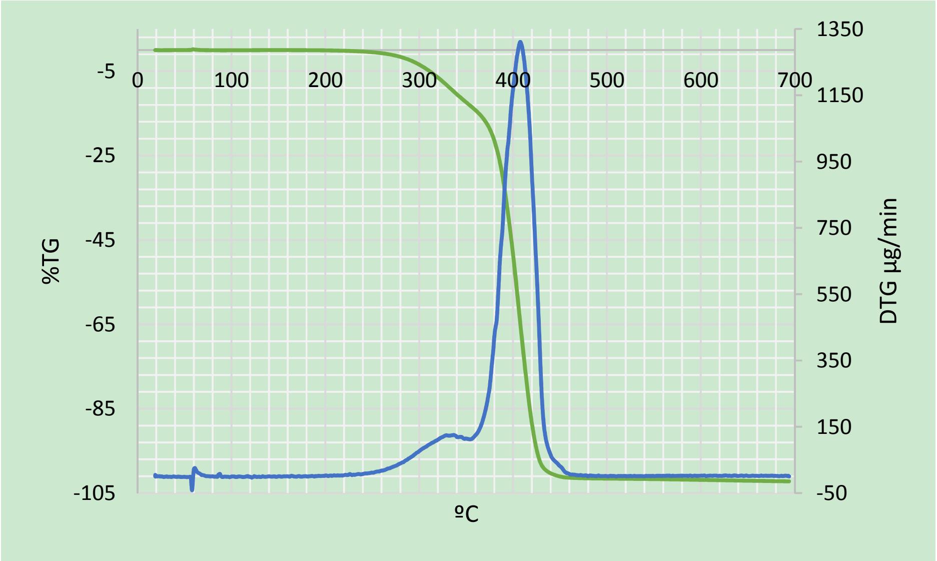

Figure S28. TGA of polymer obtained with initiator **2**

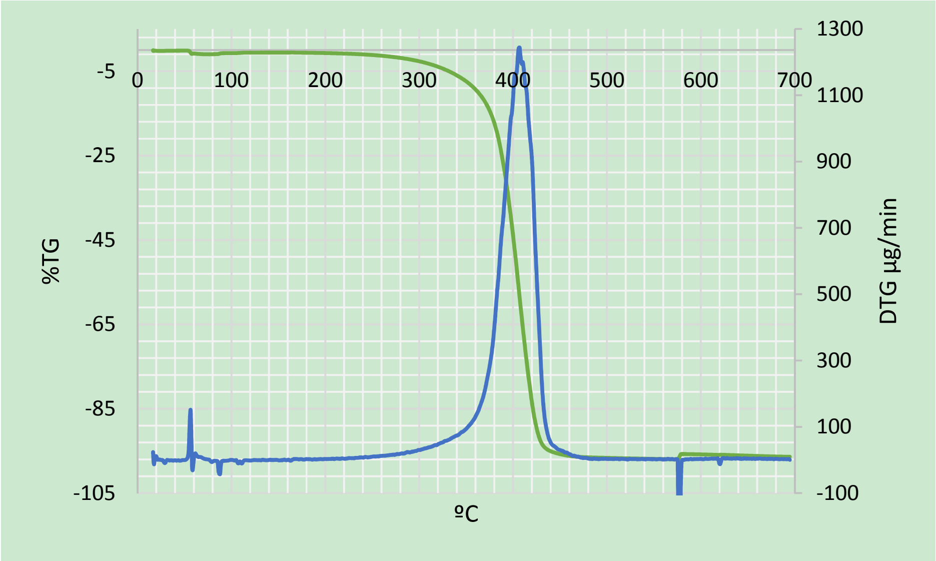

Figure S29. TGA of polymer obtained with initiator **3**

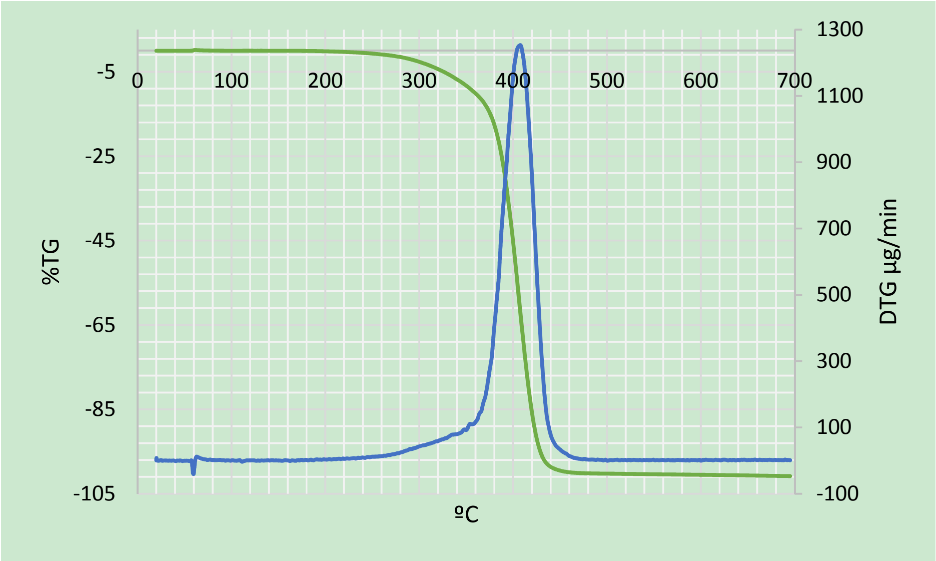

Figure S30. TGA of polymer obtained with initiator **4**

4. The Raman discussion

Some Raman spectral bands for the ligand and its CPs are shown in Table 2. The bands observed at 1377, 1254, 1143, 1066, 999 and 653 cm^{-1} can be attributed to ν(N-C)st, δ(C-H)ip and δ(C-H)oop [1-3]. The Raman spectra for these compounds have never been reported, and the bands correspond to the symmetric vibrational modes.

Table S1. Raman spectral bands for ligand 1 and its CPs.

	1	2	3	4	5
Wavenumber /cm^{-1}	178	--	172	--	135
	--	--	--	237	--
	--	--	--	304	--
	347	--	--	--	--
	402	--	--	--	
	491	--	--	--	--
	--	521	532	--	--
	--	572	572	--	--
	653	--	--	677	--
	762	--	759	758	772
	--	861	868	858	868
	998	963	--	999	963
	1066	1041	1045	1045	1041
	1143	1114	1134	1157	1119
	1254	1221	1230	1303	1221
	1377	1334	1338	1398	1336
	--	1445	1461	1496	1443

Additionally, there were some bands that were only present in the spectra of the CPs (861-868 and 1443-1496), there were some bands that shifted to lower wavenumbers with respect to the spectrum of the free ligand, and there were bands that only appeared in the spectrum of the free ligand and do not appear in the spectra of the CPs. These results are due to the coordination of the ligand to the metal.

References

1. Billes, F.; Ziegler, I.; Mikosch, H. Vibrational spectroscopic study of sodium-1,2,4-triazole, an important intermediate compound in the synthesis of several active substances. *Spectrochim. Acta Mol. Biomol. Spectrosc.* **2016**, *153*, 349–362, doi:10.1016/j.saa.2015.08.014.
2. Billes, F.; Endrédi, H.; Keresztury, G. Vibrational spectroscopy of triazoles and tetrazole. *J. Mol. Struct. THEOCHEM* **2000**, *530*, 183–200, doi:10.1016/S0166-1280(00)00340-7.
3. Bougeard, D.; Le Calvé, N.; Saint Roch, B.; Novak, A. 1,2,4-Triazole: Vibrational spectra, normal coordinate calculations, and hydrogen bonding. *J. Chem. Phys.* **1976**, *64*, 5152–5164, doi:10.1063/1.432190.
